# Effects of transcranial direct current stimulation on basketball shooting accuracy: a systematic review and meta-analysis

**DOI:** 10.3389/fspor.2026.1881913

**Published:** 2026-09-30

**Authors:** Yue Li, Xiao lu Yuan, Shuairan Li, Jie lin Yang

**Affiliations:** 1 School of Physical Education and Sports, Shenzhen University of Information Technology, Shenzhen, Guangdong, China; 2 School of Physical Education, Nanyang Normal University, Nanyang, Henan, China; 3 College of Sports Science, Tianjin Normal University, Tianjin, China; 4 South China University of Technology, Guangzhou, China

**Keywords:** basketball, meta-analysis, motor skill, neuromodulation, shooting accuracy, sports performance, transcranial direct current stimulation (tDCS)

## Abstract

**Background:**

Transcranial direct current stimulation (tDCS), a form of transcranial electrical stimulation, has been investigated as a method to enhance motor performance in sports. However, findings regarding its effects specifically on basketball shooting accuracy remain inconsistent. This systematic review and meta-analysis aimed to clarify the effects of tDCS on basketball shooting performance by synthesizing evidence from randomized controlled trials (RCTs).

**Methods:**

Following PRISMA guidelines and a pre-registered protocol (PROSPERO CRD420251066350), a comprehensive literature search was conducted across PubMed, Web of Science, EBSCO, Scopus, and CNKI. Search terms included “transcranial direct current stimulation,” “tDCS,” “basketball,” and “shooting accuracy.” Eligible studies were RCTs evaluating tDCS (anodal or cathodal) vs. sham stimulation in basketball players and reporting shooting accuracy outcomes. Two reviewers independently screened studies, extracted data, and assessed risk of bias. Meta-analysis was performed using a fixed-effect model when appropriate.

**Results:**

The pooled analysis revealed a moderate, statistically significant effect of tDCS on improving basketball shooting accuracy [pooled standardized mean difference [SMD] = 0.67, 95% confidence interval [CI] [0.43, 0.92], *p* < 0.001]. There was no significant heterogeneity among studies (*Q* test *p* = 0.288  , *I*^2^ = 17.9%), indicating no detectable between-study heterogeneity. All included studies reported improvements in shooting performance favoring tDCS over sham, though effect sizes varied. Subgroup analyses showed that the positive effect of tDCS showed beneficial effects across different shooting tasks (free throws vs. field goals), participant characteristics (including age, sex, and skill level), and stimulation parameters (current intensity: 1–2 mA; single-session vs. multi-session protocols), with no subgroup differences reaching statistical significance. Risk-of-bias assessment indicated that most studies were of moderate quality, with some limitations related to blinding and sample size.

**Conclusions:**

This meta-analysis presents a novel quantitative synthesis of existing trial evidence examining the effects of tDCS on basketball shooting performance. These preliminary findings suggest that tDCS warrants further investigation as a potential neuromodulation technique for shooting-precision training; however, given the generally small sample sizes and short follow-up durations characterizing the current evidence base, any practical application remains premature. Future research incorporating larger, ecologically valid, and longer-term study designs is needed to confirm the durability of tDCS-related benefits and to establish optimal stimulation protocols before this approach can be responsibly translated into athletic training practice.

**Systematic Review Registration:**

https://www.crd.york.ac.uk/PROSPERO/view/CRD420251066350, identifier CRD420251066350.

## 1 Introduction

Basketball shooting is a highly complex sensorimotor skill that requires precise coordination among multiple brain regions for successful execution. The shooting action involves visual localization of the basket by the occipital cortex, precise spatial trajectory planning by the parietal cortex, coordinated activation of upper-limb muscles by the motor cortex, and fine regulation of movement timing and force by the cerebellum (1, 2). Neurophysiological studies have shown that expert basketball players develop distinct neural activation patterns associated with shooting. For example, Aglioti et al. observed enhanced activity in the motor cortex mirror neuron system of elite players while observing shooting movements, reflecting neuroplastic adaptations associated with long-term training (3). Shooting accuracy is not only critical for scoring and game outcomes but also serves as a key indicator of a player's technical proficiency and psychological resilience (4). However, during intense competition, cumulative fatigue can markedly impair neural and motor function. When athletes’ heart rates approach 85%–90% of maximum, central fatigue may disrupt visuomotor coordination, leading to reduced shooting stability and accuracy (5, 6).

Transcranial direct current stimulation (tDCS) is a non-invasive brain stimulation technique that modulates cortical excitability via a weak, constant current applied through scalp electrodes (7). Anodal tDCS can modulate neuronal membrane potentials in the targeted region, thereby altering cortical excitability (8). Neurochemically, anodal tDCS is thought to modulate inhibitory GABA ergic and excitatory glutamatergic activity, thereby creating conditions that may facilitate long-term potentiation (LTP) and synaptic plasticity (9). Notably, a single 20 min tDCS session can produce after-effects lasting 60–90 min, offering a potential window of enhanced cortical excitability for motor learning and performance (10). Within motor learning research, accumulating evidence suggests that tDCS can improve a range of motor skills, including fine motor tasks such as golf putting accuracy, tennis serve consistency, and marksmanship precision (11–14).

In the context of basketball shooting, existing tDCS studies have reported mixed results. On the one hand, several trials have found that tDCS significantly improves shooting performance. Veldema et al. demonstrated in a double-blind RCT that 20 min of 1 mA anodal tDCS over the dominant motor cortex produced a significant enhancement in shooting accuracy (*p* = 0.022) and dribbling agility (*p* = 0.038) in 52 college athletes (15). Likewise, other studies have reported 10%–20% improvements in shooting success following tDCS. On the other hand, some well-controlled studies have failed to replicate these benefits. For instance, Moreira et al. applied 2 mA anodal tDCS over the left dorsolateral prefrontal cortex in professional players and found no significant change in shooting accuracy compared to sham stimulation (16). The reasons for these discrepancies are likely multifactorial. First, even subtle differences in stimulation parameters, such as current intensity (commonly 1–2 mA), stimulation duration (15–30 min), and electrode montage/placement, can lead to divergent neuromodulatory effects (17). Second, participant characteristics vary; individuals with different skill levels, training experience, ages, or sexes may respond differently to identical tDCS protocols (18). Third, inconsistencies in experimental design and outcome measures (e.g., varying shooting drills or performance metrics) make it challenging to compare results across studies. Moreover, many studies to date have methodological limitations such as small sample sizes (often <50 per study), lack of long-term follow-up, suboptimal control conditions, and incomplete blinding/randomization procedures. These issues may reduce statistical power and limit the internal validity and generalizability of the findings (19, 20).

Given the uncertainty in the literature and the practical importance of enhancing basketball shooting performance, a systematic review and meta-analysis are warranted. A systematic review uses predefined inclusion criteria, structured search strategies, and rigorous quality assessment to minimize bias and objectively evaluate the evidence base. When combined with meta-analysis, this approach quantitatively synthesizes data from multiple independent studies, providing more precise pooled effect estimates and allowing the overall body of evidence to be evaluated quantitatively. Meta-analysis also enables exploration of between-study heterogeneity and potential moderators of effect through subgroup and sensitivity analyses. In the context of basketball training, evidence-based evaluation is particularly important as novel interventions like tDCS emerge as potential adjuncts to conventional training approaches. Therefore, the present study systematically gathered all available RCT evidence on the effects of transcranial electrical stimulation (primarily tDCS) on basketball shooting accuracy. Our goals were to determine the overall magnitude and consistency of the effect of tDCS on shooting performance and to identify key factors (e.g., stimulation parameters, and player characteristics) that might influence outcomes. We anticipate that the findings will clarify the current evidence regarding the potential role of tDCS in basketball skill training and provide methodological and theoretical insights for future neuromodulation research in sport (21, 22). Ultimately, this work aims to inform evidence-based practice and guide future research on performance enhancement strategies in competitive basketball (23, 24).

## 2 Methods

The review was conducted in accordance with the PRISMA (Preferred Reporting Items for Systematic Reviews and Meta-Analyses) guidelines and was prospectively registered in PROSPERO (RegistrationNo. CRD420251066350) to ensure methodological transparency. All screening, extraction, and analyses were completed in accordance with a predefined protocol.

### 2.1 Literature search

A comprehensive literature search was performed to identify relevant studies on tDCS and basketball shooting accuracy. The following electronic databases were searched: PubMed, Web of Science, EBSCO, Scopus, and CNKI (China National Knowledge Infrastructure). The search spanned from each database's inception to July 8^th^, 2026. No language restrictions were applied, and both English- and Chinese-language sources were considered.

The search strategy combined terms related to transcranial electrical stimulation and basketball performance. Key search terms included: “transcranial direct current stimulation,” “tDCS,” “transcranial electrical stimulation,” “basketball,” “shooting accuracy,” “shooting performance,” and “skill.” These terms were used in various Boolean combinations (e.g., tDCS AND basketball, transcranial direct current stimulation AND shooting). Where appropriate, MeSH terms truncation operators were also employed to maximize the retrieval of relevant literature. For example, in PubMed, the term “basketball” was paired with related terms such as “shot” or “throw” to broaden the search.

In addition to database searches, the reference lists of all included studies and pertinent review articles were hand-searched to identify additional eligible studies not captured by the database queries. Conference proceedings and grey literature were not systematically searched due to practical constraints. The complete search strategy for each database is provided in Supplementary Table S1 (which details the exact queries and filters used).

### 2.2 Inclusion and exclusion criteria

Study eligibility was defined *a priori* based on the PICOS framework (Population, Intervention, Comparison, Outcomes, Study design). Population: studies involving basketball players or individuals practicing basketball shooting (with no restrictions on age, sex, or skill level). Intervention: transcranial electrical stimulation (tES), specifically transcranial direct current stimulation (tDCS) applied via scalp electrodes. Any tDCS montage, intensity, and duration were acceptable, provided the intervention was intended to modulate neural activity relevant to motor performance. Comparison: a sham stimulation condition or a control group performing an equivalent training protocol without active current. Outcomes: quantitative measures of basketball shooting performance (e.g., shooting accuracy percentage, free-throw or field-goal success rate, or scores on standardized shooting tests). Studies were required to report at least one objective shooting accuracy outcome. Study Design: randomized controlled trials (including crossover designs) were included.

We excluded observational studies, single-group pre-post studies without a control condition, and review articles. If multiple reports were derived from the same experiment, only the most complete and relevant report was included. Basic characteristics of the included studies are summarized in Table 1.

**Table 1:** Basic characteristics of the included studies.

Author/year	Sample characteristics	Stimulation parameters	Shooting distance
Veldema et al. (15)	Male, *n* = 19; Female, *n* = 33, Age: 22.3 ± 2.0	Participants underwent a single 20 min session of anodal tDCS (1 mA; target site unspecified) or sham stimulation of equivalent duration.	2ps
Naghizadeh et al. (25)	Male, *n* = 13; Age: 16.54 ± 3.54	Participants underwent a 3-day intervention period. The experimental group received one daily 20 min session of tDCS (1.5 mA) targeting the premotor cortex (electrode montage not specified in the original source); controls underwent daily sham tDCS of the same duration.	2ps
Naghizadeh et al. (26)	Male, *n* = 13; Age: 16.55 ± 3.5	Participants received a single 20 min session of anodal tDCS (1.5 mA) concurrent with point-light action observation learning, followed immediately by a free-throw test; controls underwent an identical protocol with sham stimulation.	FT
Mehdipour et al. (27)	Female, *n* = 15; Age: 18–24	Participants received over a 6-day intervention period, the experimental group received one daily 15 min session of tDCS (1.5 mA) targeting the motor or visual cortex.	FT
Bahrami et al. (28)	Male, *n* = 9;Age: 16.54 ± 3.54	Participants received a single 25 min session of tDCS (1.5 mA; target site and electrode montage unspecified); controls underwent sham stimulation, terminated 30 s after onset.	3ps
Mahdipour et al. (29)	Female, *n* = 15, Age: 24.4 ± 3.12	Participants received one daily 15 min session of tDCS (1.5 mA; anode at C3, cathode at Fp2) delivered concurrently with observation of a skilled-model demonstration over a 5-day intervention period, followed by 15 basketball free throws; controls observed the same model while receiving 15 min of sham stimulation.	FT
Zhang et al. (30)	Male, *n* = 20; Female, *n* = 9; Age: 19.79 ± 0.77	Participants received bilateral anodal tDCS (2 mA) over the primary motor cortex (M1) for a single 10 min session, or 30 s of sham stimulation; all participants first underwent an incremental-load cycling protocol to induce fatigue before receiving the stimulation.	FT
Liu et al. (31)	Male, *n* = 15; Age: 19.20 ± 0.57	Participants received three weekly sessions of tDCS (2 mA, 20 min per session; target site unspecified) over an 8-week intervention period.	2ps

tDCS, transcranial direct-current stimulation; C3-Fp2, international 10–20 EEG electrode sites (anode at C3, cathode at Fp2); M1, primary motor cortex; FT, free throw; 2 PS, two-point shots; 3 PS, three-point shots.

### 2.3 Data extraction and coding

All references identified from the searches were imported into EndNote X20 for deduplication and management. Two reviewers (Li and Li) independently screened titles and abstracts according to the inclusion criteria. For studies meeting the inclusion criteria or for which relevance was unclear, full-text articles were retrieved and assessed. Any disagreements in study selection were resolved through discussion or arbitration by a third reviewer (Yang), in line with PRISMA recommendations. The study selection process is illustrated in the PRISMA flow diagram (Figure 1).

**Figure 1:**
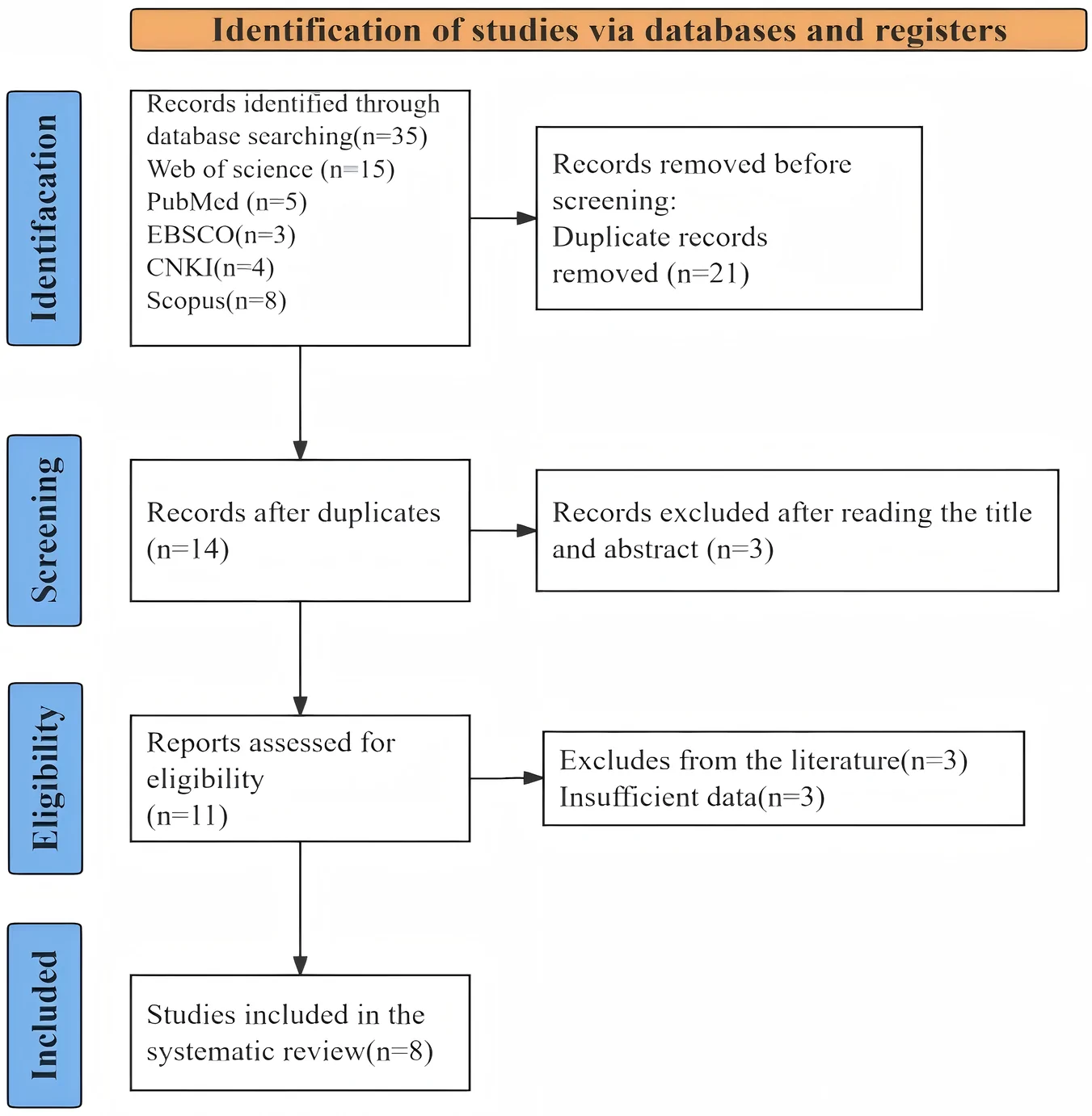
PRISMA flow diagram of the literature screening process.

For each study that met the inclusion criteria, data were extracted using a standardized form. The following information was recorded: (1) Study characteristics: authors, year of publication, country, and study design (e.g., parallel-group RCT or crossover design). (2) Participant characteristics: sample size per group, total sample size, age range/mean, sex distribution, and athletic level (e.g., elite athletes, college players, or recreational players). (3) Intervention details: tDCS montage and target area [e.g., primary motor cortex [M1] or dorsolateral prefrontal cortex [DLPFC]], current intensity (in mA), electrode size and placement (anode/cathode positions), stimulation duration (minutes per session), number of stimulation sessions, and whether tDCS was combined with any concurrent task (such as motor practice or observational learning). (4) Outcome measures: the specific metrics used to quantify shooting performance (e.g., free-throw percentage, number of shots made out of attempts, or scores on a standardized shooting drill) and the timing of outcome assessment (e.g., immediate post-stimulation, or a retention test after a delay). If studies reported multiple relevant outcomes or time points, all were noted, but priority was given to the primary shooting accuracy outcome at the earliest post-intervention time point for the primary meta-analysis. (5) Results: outcome data for shooting accuracy (means, standard deviations, or change scores for the tDCS and control groups). When results were only presented graphically, numerical values were extracted using Web Plot Digitizer (version 4.5). (6) Risk-of-bias indicators: details relevant to randomization, blinding, dropouts, and any other potential biases were collected to inform quality assessment.

We attempted to contact the study authors if any critical data were missing or unclear. In cases where authors did not respond or the required data were unavailable, those studies were included in the qualitative synthesis but omitted from the quantitative meta-analysis for the affected outcome to avoid introducing bias through data imputation.

### 2.4 Risk of bias assessment

We evaluated the risk of bias of included studies using the Cochrane Risk of Bias 2.0 tool for randomized trials. This instrument assesses potential bias across five domains: (1) bias arising from the randomization process, including the adequacy of sequence generation and allocation concealment to prevent selection bias; (2) bias due to deviations from intended interventions, including whether participants, personnel, and coaches were blinded appropriately to mitigate performance bias; (3) bias due to deviations from intended interventions, including the extent to which incomplete outcome data or dropouts were handled (e.g., with intention-to-treat analysis) to avoid attrition bias; (4) bias in measurement of the outcome, including the objectivity of outcome measurement and blinding of outcome assessors where applicable; and (5) bias in selection of the reported result, including whether reported analyses and outcomes were consistent with those specified in the study methods or protocol.

Two reviewers independently rated each domain for each study as “low risk,” “some concerns,” or “high risk.” Disagreements were resolved by consensus or by consulting a third reviewer. The results of the risk of bias assessment were presented as both a summary graph and an itemized breakdown (Figure 2). These assessments were used to inform interpretation of the certainty and methodological limitations of the included evidence.

**Figure 2:**
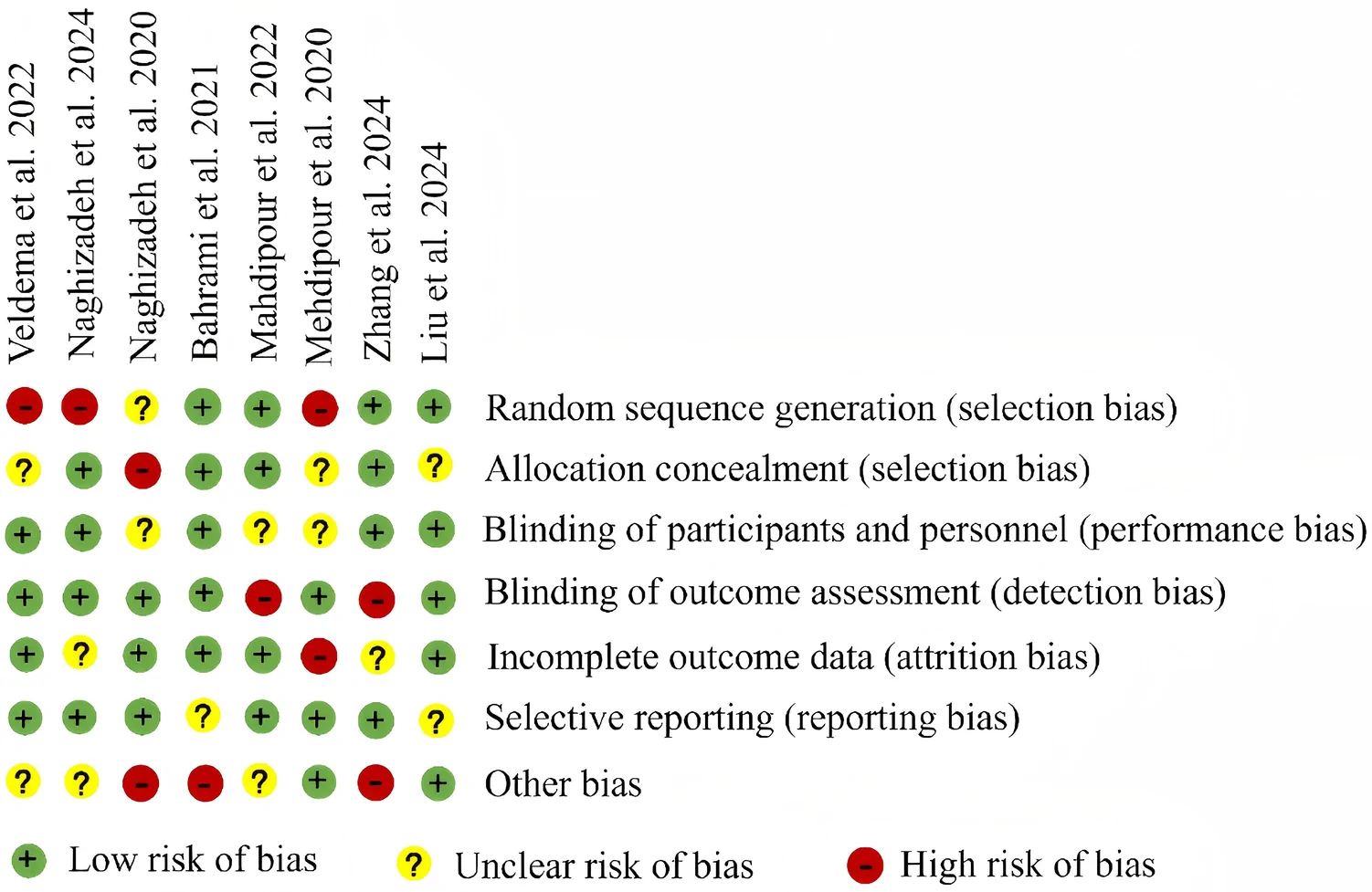
Risk of bias assessment for individual studies. Each included study is rated as low (green), unclear (yellow), or high (red) risk of bias across seven domains: randomization, allocation concealment, blinding of participants/personnel, blinding of outcome assessment, incomplete outcome data, selective reporting, and other biases.

### 2.5 Statistical methods

Outcome data (continuous measures of shooting accuracy) from each trial were pooled using meta-analytic techniques. We calculated effect sizes as standardized mean differences (SMDs) with 95% confidence intervals (CIs) for the difference between the tDCS and control (sham) groups (Table 2). Given the likelihood of variability in study populations, stimulation protocols, and outcome measures, we first assessed statistical heterogeneity using Cochran's *Q* test and the *I*^2^ statistic. For the *Q* test, a significance level of *p* < 0.10 was used because of its relatively low statistical power when few studies are available, and *I*^2^ values were interpreted as indicating increasing levels of between-study heterogeneity, with values of approximately 25%, 50%, and 75% conventionally representing low, moderate, and high heterogeneity, respectively.

**Table 2:** Subgroup analysis of the effect of transcranial electrical stimulation on basketball shooting accuracy.

Intervention variable	Sub	*K*/*N*	Meta-analysis					
			SMD	(95% CI)	*P*	*I*^2^ (%)	*Q*(df)^betw^	*P* ^betw^
Shooting distance	Two-point shot	3/54	0.657	[0.320, 0.994]	0.001	45.500	*Q_b_* (2) = 0.050	0.828
	Free throw	4/126	0.654	[0.111, 1.197]	0.008	3.600		
Age	Adult	6/109	0.958	[0.247, 1.669]	<0.001	15.800	*Q_b_* (1) = 1.060	0.303
	Adolescents	2/26	0.607	[0.305, 0.909]	0.008	34.000		
Gender	Male	4/50	1.094	[0.671, 1.517]	<0.001	0.000	*Q_b_* (2) = 2.360	0.125
	Female	2/30	0.571	[0.056, 1.086]	0.030	0.000		
Athletic level	Athlete	6/105	0.774	[0.400, 1.148]	<0.001	0.00	*Q_b_* (1) = 0.200	0.656
	Ordinary person	2/30	0.571	[0.056, 1.086]	0.030	39.000		
Session	1–3 per	5/90	0.688	[0.301, 1.075]	0.001	36.200	*Q_b_* (1) = 0.030	0.854
	5–6 per	2/30	0.571	[0.056, 1.086]	0.030	0.000		
Current intensity	1–1.5 mA	6/181	0.780	[0.343, 1.017]	0.001	18.400	*Q_b_* (1) = 0.050	0.821
	2 mA	2/44	0.794	[0.086, 1.502]	0.001	0.080		
Duration of intervention	10-15 min	3/59	0.530	[0.163, 0.897]	0.005	0.000	*Q_b_* (1) = 0.580	0.445
	20-25 min	5/76	0.807	[0.288, 1.326]	0.001	20.600		

SMD, standardised mean difference; K, number of studies; N, sample size.

In the absence of significant heterogeneity (*Q p* > 0.10 and *I*^2^ < 50%), a fixed-effect model (inverse-variance weighting) was applied. If moderate or high heterogeneity was detected, we planned to use a random-effects model (DerSimonian-Laird method) and explore potential sources of heterogeneity through subgroup analyses. Meta-regression was considered only if a sufficient number of studies were available. Given the relatively small sample sizes common in these studies, we applied the Hedges’ g bias correction to the SMDs to account for small-sample bias. Conventionally, SMDs were interpreted as approximately 0.2 = small, 0.5 = medium, 0.8 = large, and >1.0 = very large effect sizes.

We conducted subgroup analyses to examine whether the effect of tDCS on shooting accuracy differed by key characteristics: shooting task type (free throws vs. field shots), number of tDCS sessions (1–3 sessions vs. 5–6 sessions), stimulation intensity (low: 1–1.5 mA vs. high: 2 mA), player skill level (athletes vs. recreational players), age group (adult vs. adolescent), and participant sex (male vs. female). These subgroup divisions were defined *a priori*. We used the *Q* statistic to test for subgroup differences (*Q*_between), with a *p* < 0.05 indicating a significant moderator effect.

We also performed sensitivity analyses to assess the robustness of the pooled results. Each study was omitted in turn to determine whether any single study unduly influenced the pooled effect. Additionally, we compared fixed-effect and random-effects estimates to assess whether the overall conclusions were consistent across modeling assumptions.

Publication bias was assessed visually by inspecting a funnel plot of the effect size against standard error for asymmetry (Figure 3). Given that fewer than 10 studies were included, we refrained from using Egger's or Begg's formal tests because these tests have limited statistical power when the number of studies is small. The trim-and-fill method was used only as an exploratory sensitivity analysis, and its results were interpreted cautiously given the small number of included studies.

**Figure 3:**
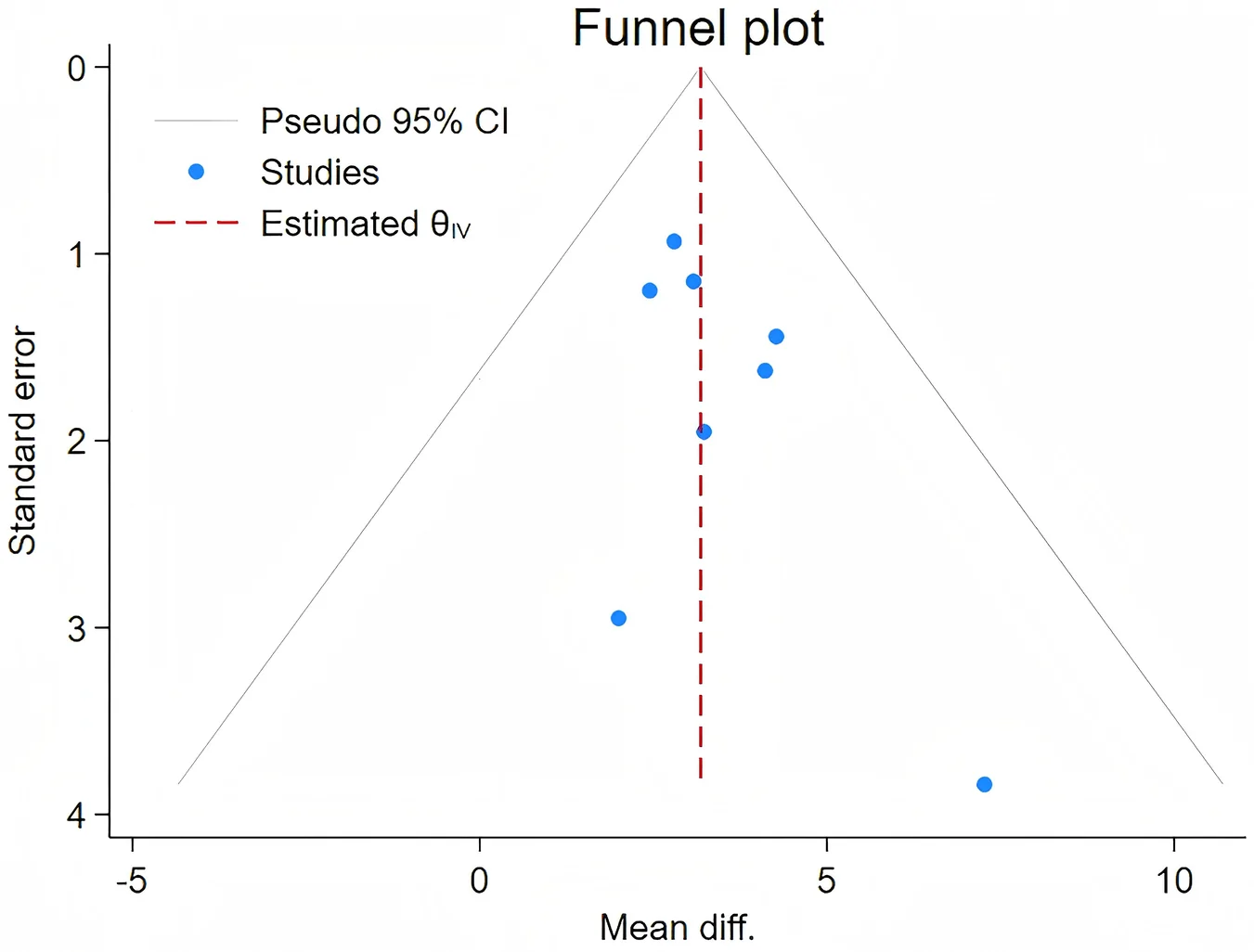
Funnel plot for assessment of potential publication bias.

All statistical analyses were conducted using Stata MP 16 and RevMan 5.4, with significance set at *p* < 0.05 for all tests except heterogeneity, as noted. The inverse-variance fixed-effect calculations were based on the following formulas:

1. The weight assigned to each effect size was calculated as: $w_{i}=\frac{1}{SE_{i}^{2}}$ (where $SE_{\dot{i}}\;$ is the standard error of Hedges’ *g* estimate for study i).
2. The standard error calculation formula for Hedges’ g is: $SE(g)=\sqrt{\frac{n_{1}+n_{2}}{n_{1}n_{2}}+\frac{g^{2}}{2(n_{1}+n_{2})}}$
3. The pooled effect size was calculated as: ${\hat{g}}_{\mathrm{combined}}=\frac{\sum_{i}w_{i}g_{i}}{\sum w_{i}}$
4. The variance and standard error of the pooled effect were calculated as: $V_{\mathrm{ar}}({\hat{g}}_{\mathrm{combined}})=\frac{1}{\sum_{i}w_{i}},\;SE=\sqrt{V_{\mathrm{ar}}}$

## 3 Results

### 3.1 Literature search results

The database search identified 35 records, and one additional record was identified through reference-list screening (Figure 1). After the removal of 21 duplicate records, 14 records remained for title and abstract screening, of which three were excluded. Eleven full-text articles were subsequently assessed for eligibility. Three articles were excluded at the full-text stage, and a further three were excluded because sufficient data for quantitative synthesis were unavailable. Ultimately, eight studies were included in the systematic review and quantitative meta-analysis.

The eight included studies met the predefined eligibility criteria and contributed to the qualitative and quantitative syntheses. All eight were randomized controlled trials published between 2020 and 2026. The total sample across these studies was more than 200 participants, with individual study sample sizes ranging from 26 to 52.

### 3.2 Characteristics of included studies

#### 3.2.1 Study and participant characteristics

The eight included RCTs were conducted in diverse geographical settings across Europe, Asia, and the Middle East. Six studies were parallel-group RCTs with separate tDCS and sham groups, whereas two used crossover designs in which participants received both tDCS and sham in counterbalanced order. Participants ranged from novice or recreational players to highly trained basketball athletes and included both adolescents and adults. Four studies included competitive athletes (e.g., university or professional team players), while the other four involved recreational or novice players. Mean participant ages generally ranged from the late teens to the mid-20s. Reflecting the athletic populations under study. Four studies enrolled exclusively or predominantly male participants, two examined female athletes, and two used mixed-gender samples. Most studies had modest sample sizes of approximately 15–30 participants per group.

#### 3.2.2 Intervention parameters

All included studies examined anodal tDCS compared with sham stimulation. The primary tDCS target in six of the eight studies was the primary motor cortex (M1), usually over the dominant hemisphere corresponding to the motor representation of the shooting arm. Typical montages place the anodal electrode over the scalp location C3 or C4 (10–20 EEG system) and the cathodal electrode on the forehead (such as the contralateral forehead or above the orbit).

One study used a different target: Moreira et al. applied stimulation to the left dorsolateral prefrontal cortex (DLPFC) to investigate effects related to mental fatigue; no significant improvement in shooting accuracy was observed (16). Another study combined motor cortex tDCS with an observational training paradigm to investigate the potential combined effects of stimulation and observational learning (15).

#### 3.2.3 Stimulation intensity and duration

Current intensities ranged from 1.0 mA to 2.0 mA. Six studies used 1.0–1.5 mA, while two studies used 2.0 mA stimulation. Stimulation duration ranged from 10 to 25 min per session, with 20 min being the most common duration. Electrode sizes varied (typically 25–35 cm^2^). Sham protocols generally involved a brief period of active current at the beginning of stimulation to reproduce the initial sensations associated with active tDCS, followed by discontinuation of current for the remainder of the session, with the aim of maintaining participant blinding.

#### 3.2.4 Intervention frequency

The included studies varied in intervention frequency. Several studies examined the effects of a single or a small number of tDCS sessions, whereas others evaluated repeated stimulation protocols. For example, one study administered tDCS before practice on five consecutive days and another implemented tDCS alongside training over an 8-week intervention period. The influence of intervention frequency on pooled effect estimates is reported in the subgroup analysis.

#### 3.2.5 Outcome measures

All studies quantified basketball shooting accuracy as a primary outcome, though the specific assessment protocols varied. Free-throw shooting performance was assessed in four studies, typically as the percentage of successful free throws from and a set number of attempts (e.g., 10 or 20 shots) or a standardized free-throw test score. Field shooting performance was evaluated in the other studies using either a “two-point” shooting drill or composite field shooting tests.

For instance, one study employed a 100-shot field goal test involving shots from multiple mid-range positions (15). Another used the standardized AAHPERD basketball shooting test, which involves a sequence of shots from different court positions (16). Some studies also measured dynamic shooting performance under fatigue or time pressure (e.g., a one-minute timed shooting drill or jump shots off the dribble).

All outcome metrics were objective counts or percentages of successful baskets, and were standardized for quantitative synthesis using SMDs. In most cases, performance was measured immediately after the tDCS or sham intervention (acute effect). A few studies additionally included retention tests at later time points (ranging from 15 min to several days post-intervention) to examine whether changes in performance were retained; those data were reported qualitatively but were not pooled in the primary meta-analysis due to heterogeneity in timing.

#### 3.2.6 Summary of study findings

The direction of the effect estimate favored tDCS over the control condition in all eight included studies. Five of the eight studies reported statistically significant improvements in their primary shooting outcome with tDCS, while the remaining three reported effect estimates favoring tDCS that did not reach statistical significance. No included study reported an effect estimate indicating poorer shooting performance following tDCS than following the control condition. Although the magnitude and statistical precision of individual effect estimates varied, their directions were consistent. Potential differences according to participant and intervention characteristics were examined formally in the subgroup analyses (Section 3.5).

### 3.3 Risk of bias assessment

The present meta-analysis included a total of eight studies: three investigated acute intervention effects, and five examined chronic intervention effects. The pooled sample comprised 270 participants (139 males and 131 females). Shooting accuracy was tested across three distances: the free-throw line, mid-range, and the three-point line. Across all studies, shooting accuracy was operationalized as the ratio of made shots to total shot attempts. Methodological quality assessment was performed following the protocols outlined in Section 2.4. One study was classified as having some concerns regarding risk of bias, while seven studies were deemed to be at high risk of bias. The results of the risk-of-bias assessment are summarized in Figure 2.

### 3.4 Meta-analysis

#### 3.4.1 Meta-analysis of the effects of tDCS on basketball shooting accuracy

Eight studies were included in the meta-analysis of the effect of tDCS on basketball shooting accuracy (Figure 4). Effect sizes were pooled using an inverse-variance fixed-effect model. The pooled SMD was 0.67 (95% CI: 0.43–0.92, *z* = 5.35, *p* < 0.001), indicating a moderate-magnitude beneficial effect of tDCS relative to sham stimulation. Heterogeneity across studies was low, with (*I*^2^ = 17.9%, *H*^2^ = 1.22), indicating that low heterogeneity existed across the included studies.

**Figure 4:**
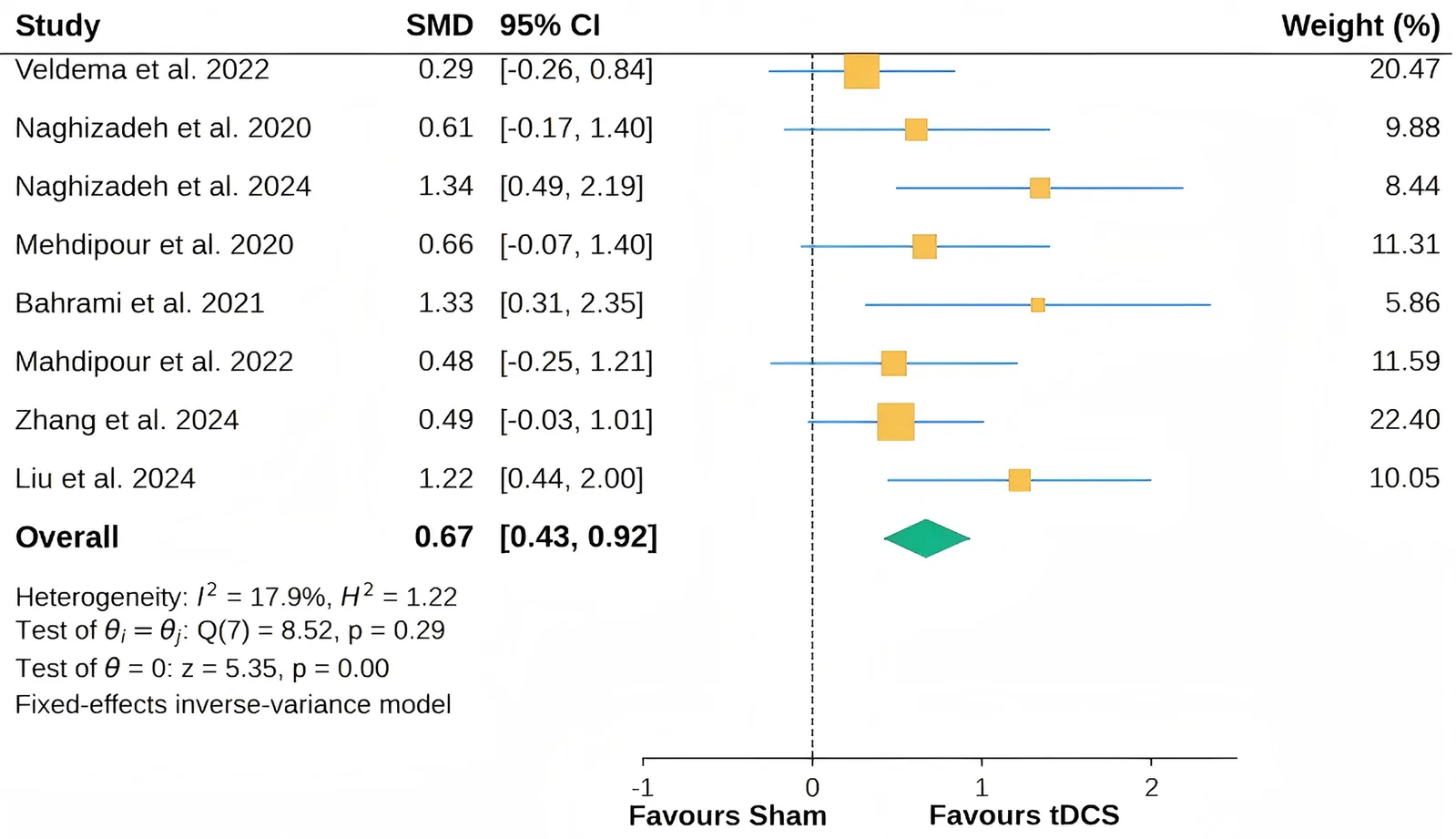
Forest plot of meta-analysis on the effect of tDCS on basketball shooting accuracy.

Overall, the pooled analysis showed a moderate effect estimate favoring tDCS, although this estimate should be interpreted in the context of the small number and sample sizes of the included trials.

### 3.5 Subgroup analysis of transcranial electrical stimulation effects on shooting accuracy

For shooting task, the pooled SMD was 0.657 (95% CI: 0.320–0.994) for two-point shots and 0.654 (95% CI: 0.111–1.197) for free throws. No statistically significant subgroup difference was detected (*Qb* = 0.005, *p* = 0.828).

Regarding participant age, the pooled SMD was 0.958 (95% CI: 0.247–1.669) for adults and 0.607(95% CI: 0.305–0.909) for adolescents, with no evidence of a subgroup difference (*Qb* = 1.060, *p* = 0.303). For participant gender, the pooled SMD was 1.094 (95% CI: 0.671–1.517) for males and 0.571 (95% CI: 0.056–1.086) for females. No statistically significant subgroup difference was detected (*Qb* = 2.360, *p* = 0.125). Regarding athletic level, pooled SMDs were 0.774 for athletes and and 0.571 for non-athletes, with no statistically significant subgroup difference (*Qb* = 0.200, *p* = 0.656). Regarding intervention frequency, pooled SMDs were 0.688 for 1–3 sessions and 0.571 for 5–6 sessions, with no statistically significant subgroup difference (*Qb* = 0.030, *p* = 0.854). For current intensity, pooled SMDs were 0.780 for 1–1.5 mA and 0.794 for 2 mA, with no evidence of a subgroup difference (*Qb* = 0.050, *p* = 0.821). For intervention duration, pooled SMDs were 0.530 (95% CI: 0.163–0.897) for 10–15 min and 0.807 (95% CI: 0.288–1.326) for 20–25 min. The between-subgroup difference was not statistically significant (*Qb* = 0.580, *p* = 0.445). None of the prespecified tests for subgroup differences reached statistical significance (all *p* > 0.05). Thus, within the limitations of the available data, there was no statistical evidence that shooting task, age, sex, athletic level, intervention frequency, stimulation intensity, or stimulation duration modified the estimated effect of tDCS on shooting accuracy.

### 3.6 Publication bias

Potential publication bias was assessed visually using a funnel plot (Figure 3). The plot showed some asymmetry, particularly among studies with larger standard errors. However, because only eight studies were included, visual interpretation of funnel-plot asymmetry is inherently uncertain.

Each point represents an included study. The vertical line indicates the pooled effect estimate, and the diagonal lines indicate the expected 95% confidence limits. Because only eight studies were included, the funnel plot should be interpreted cautiously. We did not perform Egger's regression or Begg's test because formal tests for funnel-plot asymmetry have limited statistical power when fewer than 10 studies are available. An exploratory trim-and-fill analysis did not impute additional studies. An exploratory trim-and-fill analysis did not impute additional studies.

These findings do not establish the absence of publication bias. Given the small number of studies and the uniformly positive direction of the observed effect estimates, the possibility of small-study effects or selective publication cannot be excluded. The funnel-plot and trim-and-fill findings should therefore be regarded as exploratory.

## 4 Discussion

### 4.1 Overall and subgroup effect findings of tDCS on basketball shooting accuracy

The present meta-analysis suggests that tDCS may improve basketball shooting accuracy, with a moderate-to-large pooled effect and effect estimates consistently favoring active stimulation across the included studies. Subgroup analyses yielded SMDs ranging from 0.420 to 0.974 across different shooting tasks, participant characteristics, and stimulation protocols. No statistically significant subgroup differences were detected for shooting task, age, sex, athletic level, stimulation intensity, intervention duration, or number of sessions. These findings suggest that the observed effect was not clearly confined to a particular experimental condition or participant subgroup. However, several subgroup estimates were based on only a small number of studies, which substantially limits the power to detect genuine moderator effects. The absence of statistically significant subgroup differences should therefore be interpreted cautiously and should not be taken as evidence that these factors are clinically or physiologically equivalent. The number of stimulation sessions is particularly relevant when interpreting the present findings. Effect estimates were similar for interventions involving 1–3 sessions and those involving 5–6 sessions (SMD = 0.688 vs. 0.571). Although this result might initially suggest that increasing the number of stimulation sessions provides little additional benefit, the available evidence does not support such a conclusion. However, this apparent equivalence must be interpreted with considerable caution. Only two studies contributed to the 5–6-session subgroup, and long-term follow-up was generally lacking. The analysis was therefore underpowered to distinguish between a true absence of a dose-response relationship and insufficient evidence to detect one. In particular, the similarity between the two subgroup estimates should not be interpreted as evidence of a physiological ceiling effect.

### 4.2 Neurophysiological mechanisms underlying tDCS-mediated motor performance improvement

Previous experimental studies nevertheless provide a plausible basis for short-term effects of tDCS on motor performance. Nitsche and Paulus (8) showed that anodal stimulation can increase motor-cortical excitability and that these effects may persist beyond the stimulation period (10). Such effects have subsequently been associated with changes in neuronal membrane excitability, inhibitory and excitatory neurotransmission, and synaptic plasticity (32). Within the domain of motor skills, Reis et al. (33) reported tDCS-related improvements in fine motor control, while Vines et al. (34) demonstrated potential facilitative effects in learning complex motor sequences (33, 34). The pooled estimate observed for the 1–3-session subgroup is directionally consistent with these findings, although its magnitude should be interpreted cautiously given the small samples of the included trials. Zhang et al. investigated the impact of tDCS stimulation patterns on primary motor cortex activity and subsequent shooting performance in basketball players experiencing exercise fatigue. They found that 10 min of bilateral tDCS at 2 mA over M1 improved mid-range jump shot accuracy in fatigued basketball players. Veldema et al. (15) reported that tDCS over M1 enhanced shooting accuracy in basketball athletes (15). These findings are biologically plausible because basketball shooting requires tightly coordinated control of the lower limbs, trunk, and upper extremities, together with precise temporal regulation of force production. M1 contributes to the planning and execution of this motor output. Waters et al. (35) reported bilateral M1 involvement during motor-skill acquisition, providing a potential neurophysiological rationale for targeting motor cortical networks during skill training (35). Nevertheless, the included basketball trials primarily assessed behavioral outcomes, and direct evidence linking changes in M1 excitability to improvements in shooting accuracy remains limited.

The lack of an apparent increase in effect size with a greater number of stimulation sessions also warrants consideration in relation to neuroplasticity. Nonlinear dose-response relationships have been proposed in the broader tDCS literature, including possible ceiling or inverted-U effects (36, 37). However, these mechanisms were not directly tested in the present dataset. From a neuroplasticity perspective, acute tDCS may influence motor performance through relatively rapid changes in membrane excitability and synaptic transmission, whereas longer-lasting effects may additionally involve NMDA-receptor-dependent plasticity, BDNF signaling, and LTP-like processes (38, 39).

Whether repeated stimulation produces cumulative neuroplastic benefits beyond repeated short-term effects cannot be determined from the current studies. Longer intervention periods and appropriately timed retention assessments will be required to distinguish these possibilities. The present findings should also be considered in the context of the broader sports literature. Acute tDCS has been investigated as a potential adjunct to athletic performance and motor learning, and Okano et al. (40) reported improved endurance performance following stimulation in cyclists (40, 41). Reis and Fritsch (42) similarly proposed the use of tDCS in temporal proximity to motor practice as a means of facilitating learning (42). These observations support continued investigation of tDCS in sports settings, but they do not yet justify its routine use in basketball training. Future studies should examine longer stimulation and follow-up periods, individual variability in responsiveness, optimization of stimulation parameters, and potential interactions between tDCS and task-specific training (43–45).

Several neurophysiological mechanisms could plausibly contribute to the observed behavioral effects of tDCS in sports (46–48). These potential mechanisms include changes in cortical excitability, synaptic plasticity, motor-control networks, and cognitive processing. Importantly, most of these mechanisms were not directly measured in the basketball studies included in the present meta-analysis.

tDCS can induce a slight shift in the resting membrane potential of cortical neurons by applying a weak direct current across two electrodes on the scalp. Under conventional stimulation conditions, anodal and cathodal stimulation may produce directionally different changes in cortical excitability, although the magnitude and direction of these effects depend on stimulation intensity, duration, montage, and individual neurophysiology (10). This modulation may involve changes in inhibitory and excitatory neurotransmission (9). Within M1, anodal stimulation has been associated with increased MEP amplitudes and altered corticospinal excitability (49). Network-level studies further suggest that tDCS can influence functional connectivity beyond the directly stimulated cortical region (50). These effects provide a plausible mechanism through which stimulation could influence motor performance, but they should not be interpreted as demonstrated mediators of the improvements observed in the present meta-analysis.

Synaptic plasticity may also contribute to longer-lasting behavioral effects. Animal studies indicate that tDCS produces effects analogous to LTP, dependent on NMDA receptor activation and increased BDNF-dependent signaling (39). Human experiments further suggest that tDCS may influence motor-skill consolidation across training sessions (33). However, the current basketball literature does not provide sufficient evidence to conclude that repeated tDCS produces durable neural reorganization or long-term retention of shooting skills. The repeated-session studies were few, and follow-up was limited.

Changes in motor control and sensorimotor integration represent another potential pathway. At the cortical level, modulation of motor cortex excitability may influence movement output and motor-unit recruitment (51). At the network level, tDCS may alter connectivity between M1 and other motor regions involved in movement preparation and execution. Cerebellar and motor-network stimulation has also been associated with changes in motor adaptation and error processing (52, 53). Naghizadeh et al. (54) reported improved free-throw performance following stimulation, but the study did not establish whether changes in sensorimotor integration mediated this behavioral effect (54). Effects of tDCS on postural control have also been reported in other experimental settings Poortvliet et al. (55), although their relevance to basketball shooting remains uncertain (55).

tDCS may influence cognitive processes relevant to motor performance through stimulation of the prefrontal cortex and its connections with cognitive control networks. Previous studies have investigated potential effects on attention, working memory, decision-making, and mental fatigue (11, 16). Experimental and sports-context evidence suggests that anodal stimulation of the left DLPFC may influence working-memory performance (56). Subsequent meta-analyses further indicate that effects on executive outcomes vary across tasks and protocols (57). Repeated DLPFC stimulation has also been examined in athletic populations for its potential effects on attention and memory-related performance (58). Such effects could theoretically influence shooting under cognitively demanding conditions, but this pathway was not directly tested in the basketball trials included here. Cathodal DLPFC stimulation has also been proposed to alter explicit cognitive control during highly practiced motor performance (12). Other studies have examined possible effects of prefrontal stimulation on affective regulation, perceived stress, and competitive anxiety (11, 59). Whether these cognitive and affective changes mediate basketball shooting performance remains uncertain.

Taken together, the effects of tDCS on basketball shooting are likely to reflect interactions among cortical excitability, synaptic plasticity, motor-control networks, and cognitive processes rather than a single mechanism. The available behavioral findings are compatible with such a multilevel framework, but the existing studies generally lack concurrent neurophysiological measurements capable of establishing mediation (16, 52).

## 5 Limitations and future research directions

Several limitations of the current evidence should be considered when interpreting the findings of this meta-analysis. The participant populations were also relatively restricted in terms of age, competitive level, and training background, which further limits the generalizability of the findings to the broader basketball population. Publication bias and small-study effects also cannot be excluded. Only eight studies were available, limiting both the interpretability of funnel-plot asymmetry and the statistical power of formal tests for publication bias. Moreover, although not all individual studies reached statistical significance, all study-specific point estimates favored tDCS. This pattern may reflect a genuine treatment effect but is also compatible with selective publication or other small-study effects. The magnitude of the pooled effect estimate also warrants particular caution. Although the direction of effect was consistent across the included studies, replication in larger and independently conducted trials is needed before the magnitude of the effect can be considered robust. Future research should therefore prioritize adequately powered, rigorously randomized, and blinded trials with clearly reported stimulation protocols and outcome definitions. Longer follow-up periods are needed to determine whether improvements persist beyond the immediate post-stimulation period and whether repeated stimulation produces cumulative benefits. Studies should report both absolute changes in shooting performance and standardized effect estimates, together with sufficient baseline and variance data to facilitate reliable effect-size estimation.

## 6 Conclusions

This systematic review and meta-analysis indicates that tDCS may yield improvements in basketball shooting accuracy; pooled analysis of eight RCTs demonstrates a moderate effect size in favor of active stimulation. Positive effect estimates were observed across different shooting tasks and participant characteristics, although the limited number and small sample sizes of the included studies warrant cautious interpretation. Although tDCS may have potential as an adjunct to conventional training, the current evidence is insufficient to support its routine application in basketball practice or competition. The available evidence is based predominantly on small trials with limited follow-up and substantial variation in stimulation protocols and experimental conditions. Consequently, the durability of the observed effects, optimal stimulation parameters, and generalizability to real-world competitive settings remain uncertain. Ethical, safety, and regulatory considerations should also be considered before broader implementation in athletic settings. Future studies should use larger and more diverse samples, directly compare stimulation protocols, incorporate ecologically valid performance outcomes, and include longer-term follow-up to determine whether any benefits are sustained. Collectively, the current findings provide preliminary quantitative evidence supporting further investigation of tDCS as a potential approach for modulating complex precision motor performance. Whether these findings extend to other sports or motor tasks requires further investigation. Until these findings are confirmed in larger, rigorously designed trials, tDCS should be regarded as an experimental neuromodulation approach rather than an established method for enhancing athletic performance.

## Data Availability

The raw data supporting the conclusions of this article will be made available by the authors, without undue reservation.
