# Supplementary material for: Effects of transcranial direct current stimulation on basketball shooting accuracy: a systematic review and meta-analysis
Source: Front Sports Act Living. 2026 Sep 30;8:1881913. doi: 10.3389/fspor.2026.1881913 (PMC13662558; doi:10.3389/fspor.2026.1881913)
Supplement: Supplementary file 1 [file Table1.docx]

Supplementary Material

[Supplementary Section S1. Search Strategies and Database Yields	1](#_Toc14861)

[S1.1 Search period	2](#_Toc22784)

[S1.2 Database search yields	2](#_Toc3725)

[S1.3 Full database-specific search strategies	2](#_Toc24809)

[S1.3.1 PubMed	2](#_Toc21054)

[S1.3.2 Web of Science	3](#_Toc16559)

[S1.3.3 EBSCO	4](#_Toc31488)

[S1.3.4 Scopus	5](#_Toc5199)

[S1.3.5 CNKI	6](#_Toc5199)

# Supplementary Section S1. Search Strategies and Database Yields

## S1.1 Search period

The five databases were searched from inception to 8 July 2026 using the predefined database-specific search strategies and eligibility criteria.

## S1.2 Database search yields

**Supplementary Table S1. Database search yields**

| **Database** | **Records identified** |
| --- | --- |
| PubMed | 5 |
| Web of Science | 15 |
| CNKI | 4 |
| EBSCO | 3 |
| Scopus | 8 |
| Total | 35 |

## S1.3 Full database-specific search strategies

## S1.3.1 PubMed

| Database | Search syntax | Records identified |
| --- | --- | --- |
| PubMed  (MeSH + Title-Abstract-Keyword)  Medline via PubMed | <br>#1 "Transcranial Direct Current Stimulation"[MeSH Terms] OR "transcranial direct current stimulation"[Title/Abstract] OR tDCS[Title/Abstract] OR "transcranial electrical stimulation"[Title/Abstract] OR tES[Title/Abstract] OR "non-invasive brain stimulation"[Title/Abstract] OR neuromodulation[Title/Abstract]<br>#2 basketball[Title/Abstract]<br>#3 "shooting accuracy"[Title/Abstract] OR "shooting performance"[Title/Abstract] OR "free-throw"[Title/Abstract] OR "jump-shot"[Title/Abstract] OR shoot*[Title/Abstract]<br>#4 "Randomized Controlled Trial"[MeSH Terms] OR randomized[Title/Abstract] OR randomised[Title/Abstract] OR trial[Title/Abstract] OR "controlled trial"[Title/Abstract] OR sham[Title/Abstract] OR crossover[Title/Abstract]<br>#5 #1 AND #2 AND #3 AND #4<br> | 5 |

## S1.3.2 Web of Science

| Database | Search syntax | Records identified |
| --- | --- | --- |
| Web of Science | TS=((("transcranial direct current stimulation" OR tDCS OR "transcranial electrical stimulation" OR tES OR "non-invasive brain stimulation" OR neuromodulation) AND basketball AND ("shooting accuracy" OR "shooting performance" OR "free-throw" OR "jump-shot" OR shoot*))) | 15 |

## S1.3.3 EBSCO

| Database | Search syntax | Records identified |
| --- | --- | --- |
| EBSCOhost  (TX = All-text expanded field) | TX((("transcranial direct current stimulation" OR tDCS OR "transcranial electrical stimulation" OR tES OR "non-invasive brain stimulation" OR neuromodulation) AND basketball AND ("shooting accuracy" OR "shooting performance" OR "free-throw" OR "jump-shot" OR shoot*)) AND (random* OR randomized OR randomised OR trial OR "controlled trial" OR control* OR sham OR crossover OR "cross-over")) | 3 |

## S1.3.4 Scopus

| Database | Search syntax | Records identified |
| --- | --- | --- |
| Scopus  (TITLE‑ABS‑KEY = title‑abstract‑keywords) | TITLE‑ABS‑KEY((("transcranial direct current stimulation" OR tDCS OR "transcranial electrical stimulation" OR tES OR "non‑invasive brain stimulation" OR neuromodulation) AND basketball AND ("shooting accuracy" OR "shooting performance" OR "free‑throw" OR "jump‑shot" OR shoot*))) | 8 |

## S1.3.5 CNKI

| Database | Search syntax | Records identified |
| --- | --- | --- |
| CNKI | Displayed for supplementary material: SU: (basketball); TKA: (“transcranial direct current stimulation” OR tDCS OR "transcranial electrical stimulation" OR “non‑invasive brain stimulation” OR neuromodulation) AND (“shooting accuracy” OR "shooting performance" OR “free‑throw” OR “jump‑shot” OR shoot)  Native Chinese search string for CNKI platform implementation:  SU=(' 篮球 ') AND TKA=(' 经颅直流电刺激 '+'tDCS'+' 经颅电刺激 '+' 无创脑刺激 '+' 神经调控 ') AND TKA=(' 投篮精度 '+' 投篮表现 '+' 罚球 '+' 跳投 '+' 投篮 ') | 4 |
